# Supplementary figures and images for: Control of ciliary transcriptional programs during spermatogenesis by antagonistic transcription factors
Source: eLife. 2025 Feb 26;13:RP94754. doi: 10.7554/eLife.94754 (PMC11864758; doi:10.7554/eLife.94754)

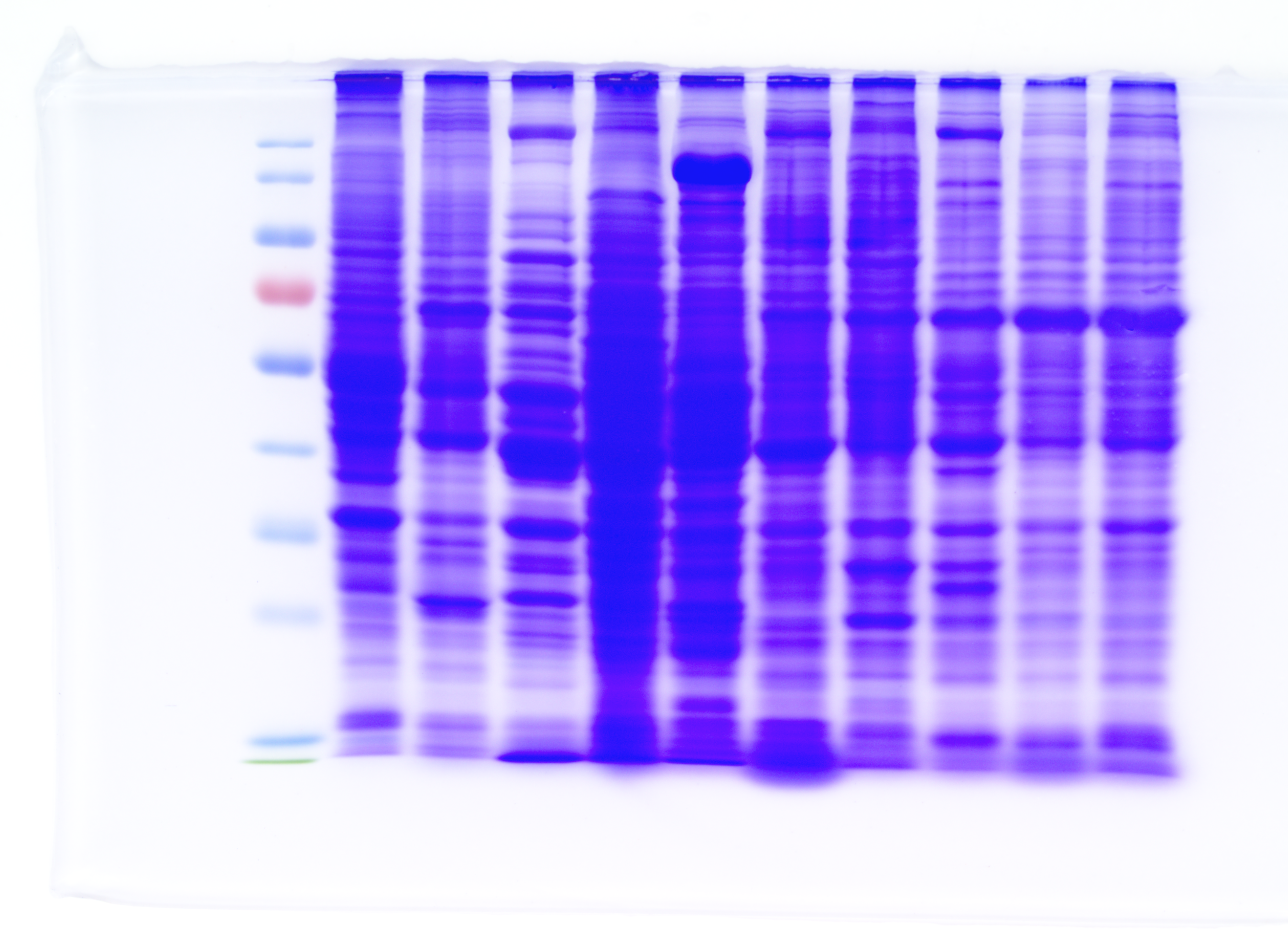

Supplement: Figure 1—source data 1. [file elife-94754-fig1-data1.zip › Figure 1—source data 1/panel A/CBB staining.tif]

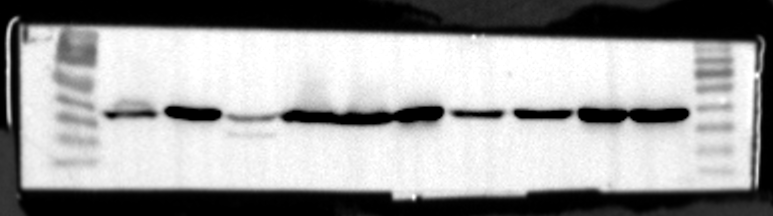

Supplement: Figure 1—source data 1. [file elife-94754-fig1-data1.zip › Figure 1—source data 1/panel A/Xap5.tif]

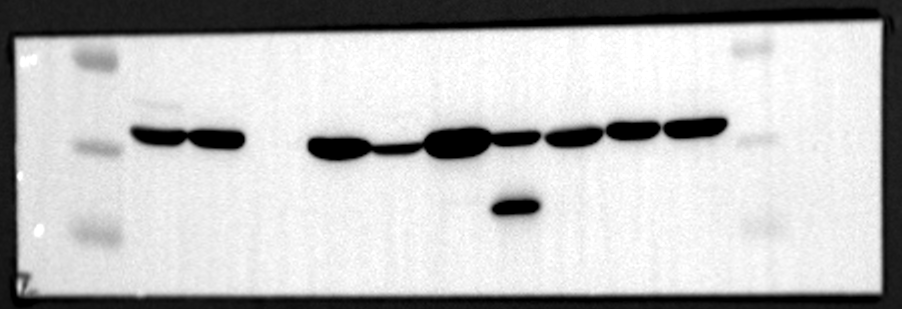

Supplement: Figure 1—source data 1. [file elife-94754-fig1-data1.zip › Figure 1—source data 1/panel A/Xap5l.tif]

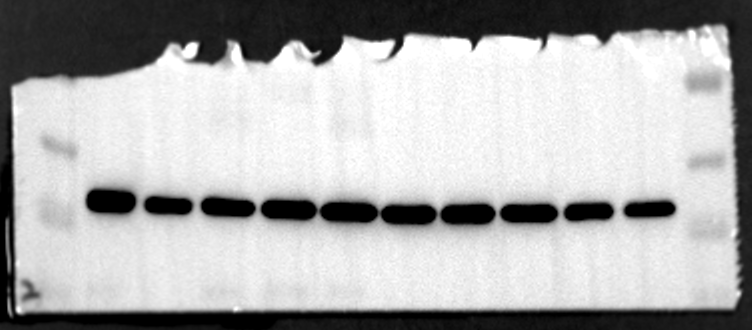

Supplement: Figure 1—source data 1. [file elife-94754-fig1-data1.zip › Figure 1—source data 1/panel B/beta-actin.tif]

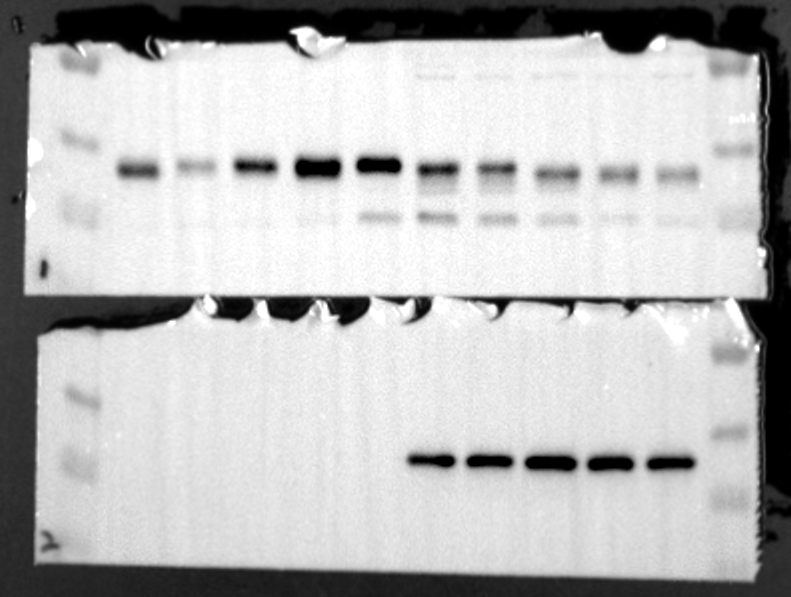

Supplement: Figure 1—source data 1. [file elife-94754-fig1-data1.zip › Figure 1—source data 1/panel B/Xap5+Xap5l.tif]

Figure 1

A

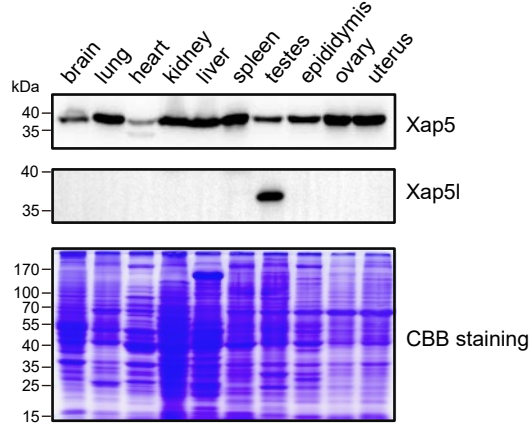

Raw blots:

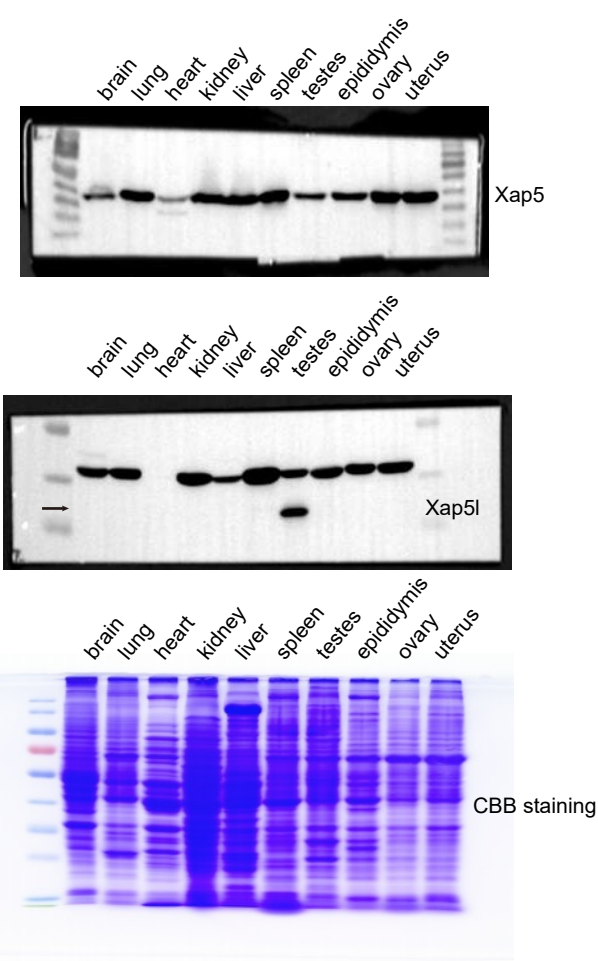

B

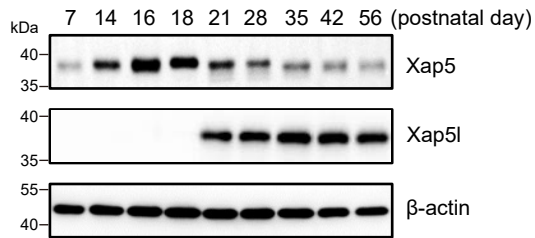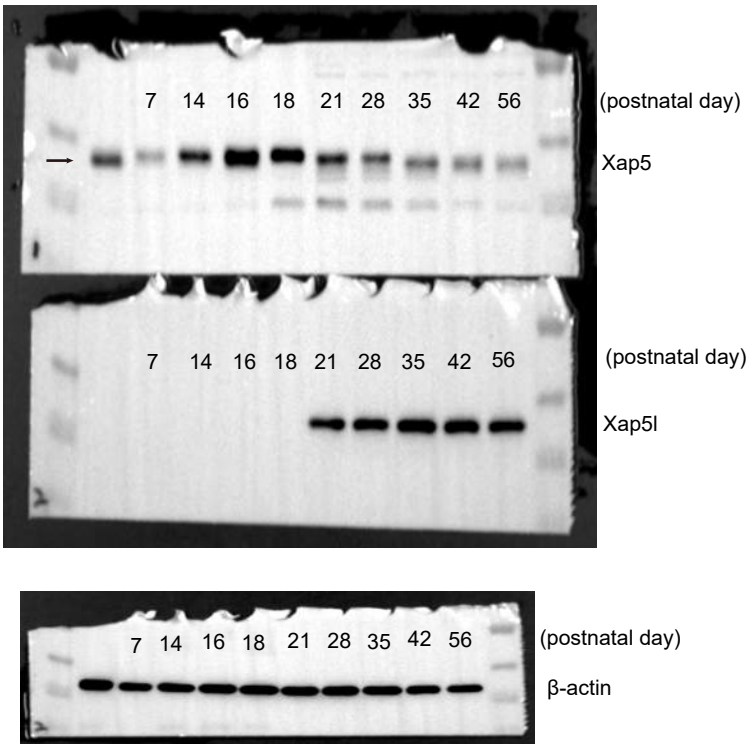

Supplement: Figure 1—source data 2. [file elife-94754-fig1-data2.zip › Figure 1—source data 2/Figure 1—source data 2.pdf]

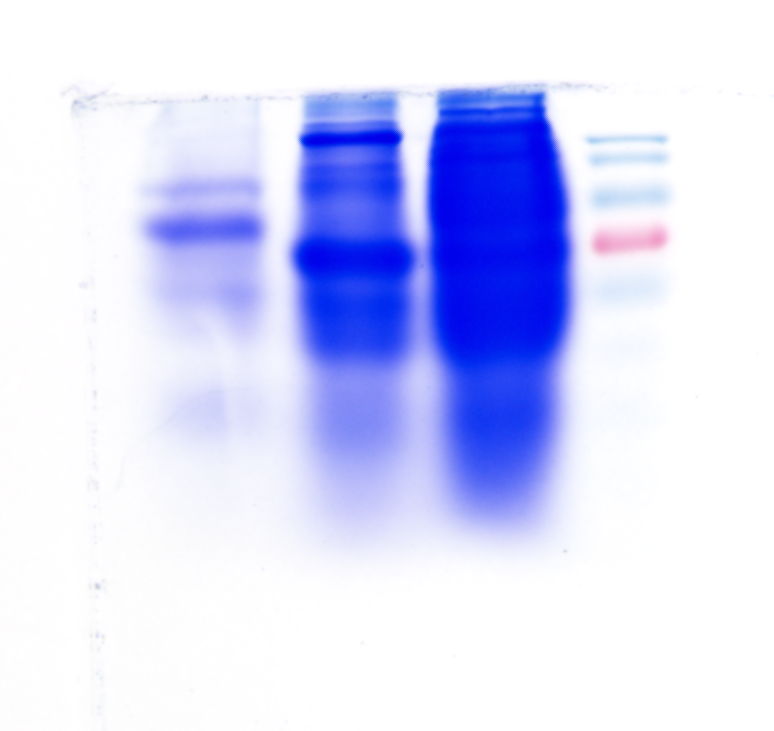

Supplement: Figure 1—figure supplement 1—source data 1. [file elife-94754-fig1-figsupp1-data1.zip › Figure 1—figure supplement 1—source data 1/panel A/CBB staining.tif]

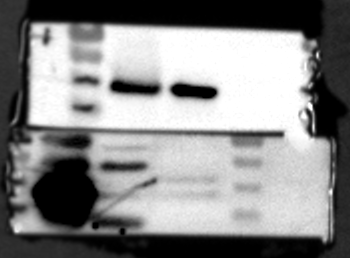

Supplement: Figure 1—figure supplement 1—source data 1. [file elife-94754-fig1-figsupp1-data1.zip › Figure 1—figure supplement 1—source data 1/panel A/Xap5.tif]

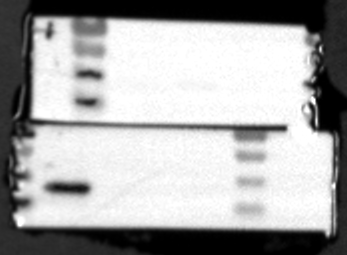

Supplement: Figure 1—figure supplement 1—source data 1. [file elife-94754-fig1-figsupp1-data1.zip › Figure 1—figure supplement 1—source data 1/panel A/Xap5l.tif]

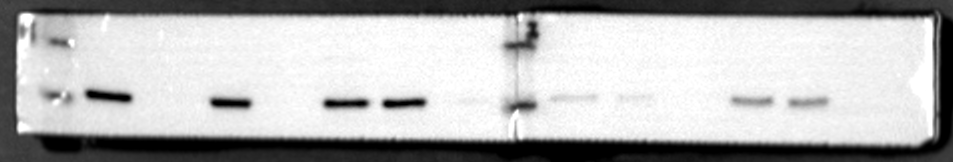

Supplement: Figure 1—figure supplement 1—source data 1. [file elife-94754-fig1-figsupp1-data1.zip › Figure 1—figure supplement 1—source data 1/panel B/Gapdh.tif]

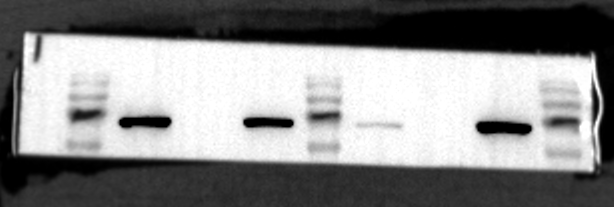

Supplement: Figure 1—figure supplement 1—source data 1. [file elife-94754-fig1-figsupp1-data1.zip › Figure 1—figure supplement 1—source data 1/panel B/Lmnb.tif]

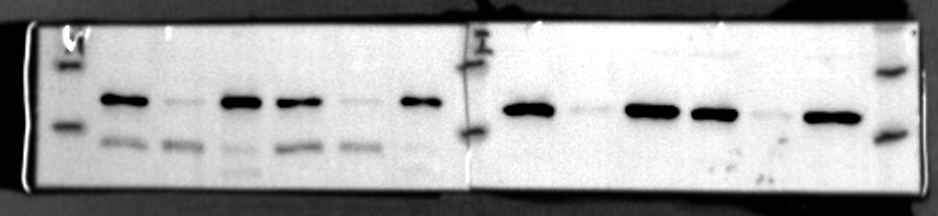

Supplement: Figure 1—figure supplement 1—source data 1. [file elife-94754-fig1-figsupp1-data1.zip › Figure 1—figure supplement 1—source data 1/panel B/Xap5.tif]

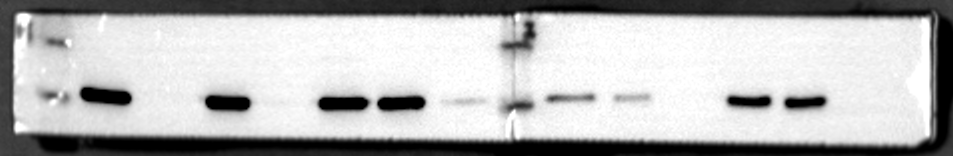

Supplement: Figure 1—figure supplement 1—source data 1. [file elife-94754-fig1-figsupp1-data1.zip › Figure 1—figure supplement 1—source data 1/panel C/Gapdh.tif]

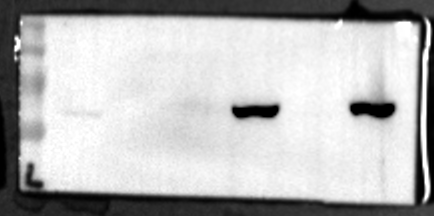

Supplement: Figure 1—figure supplement 1—source data 1. [file elife-94754-fig1-figsupp1-data1.zip › Figure 1—figure supplement 1—source data 1/panel C/Lmnb.tif]

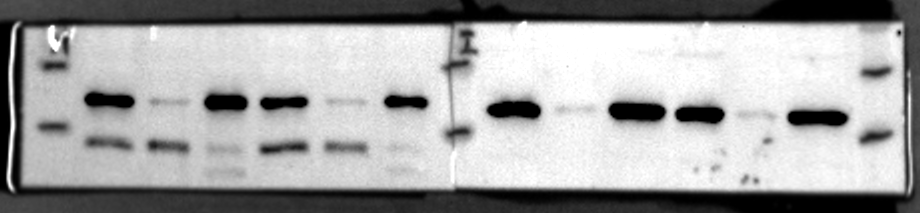

Supplement: Figure 1—figure supplement 1—source data 1. [file elife-94754-fig1-figsupp1-data1.zip › Figure 1—figure supplement 1—source data 1/panel C/Xap5l.tif]

Figure1—figure supplement 1

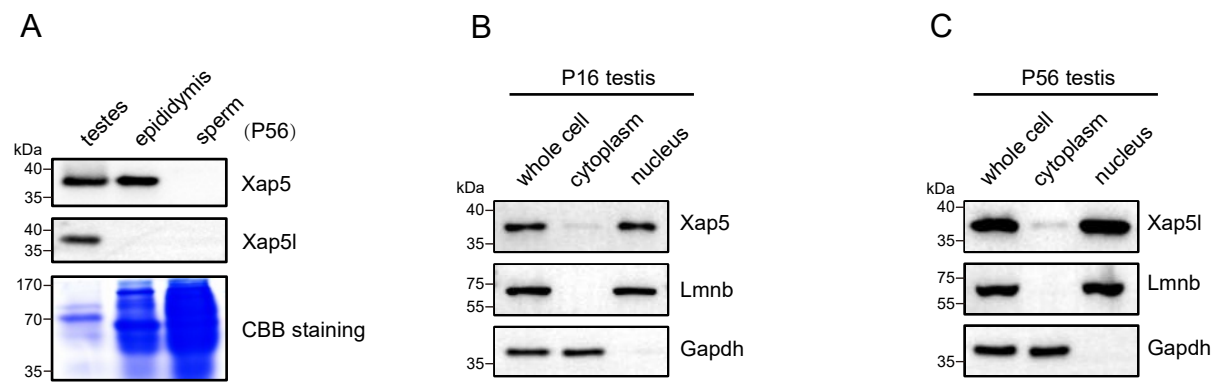

Raw blots:

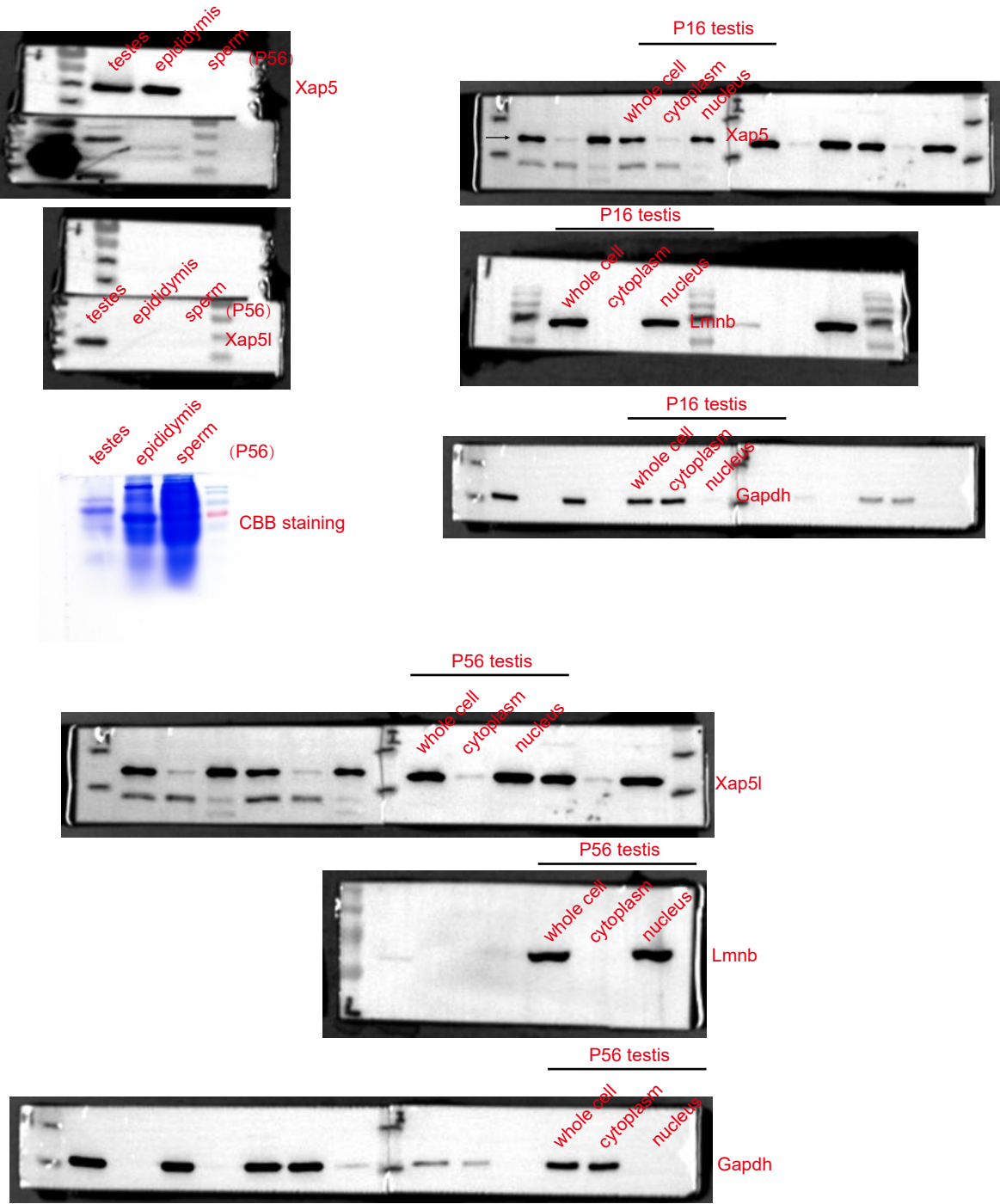

Supplement: Figure 1—figure supplement 1—source data 2. [file elife-94754-fig1-figsupp1-data2.zip › Figure 1—figure supplement 1—source data 2/Figure 1—figure supplement 1—source data 2.pdf]

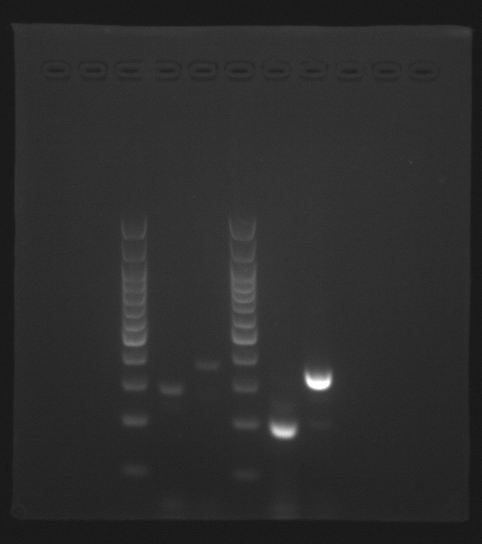

Supplement: Figure 1—figure supplement 2—source data 1. [file elife-94754-fig1-figsupp2-data1.zip › Figure 1—figure supplement 2—source data 1/panel B/B.tif]

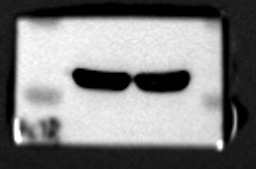

Supplement: Figure 1—figure supplement 2—source data 1. [file elife-94754-fig1-figsupp2-data1.zip › Figure 1—figure supplement 2—source data 1/panel C/beta-actin.tif]

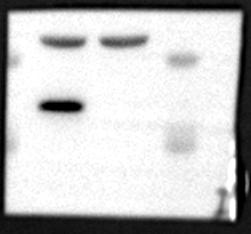

Supplement: Figure 1—figure supplement 2—source data 1. [file elife-94754-fig1-figsupp2-data1.zip › Figure 1—figure supplement 2—source data 1/panel C/Xap5l.tif]

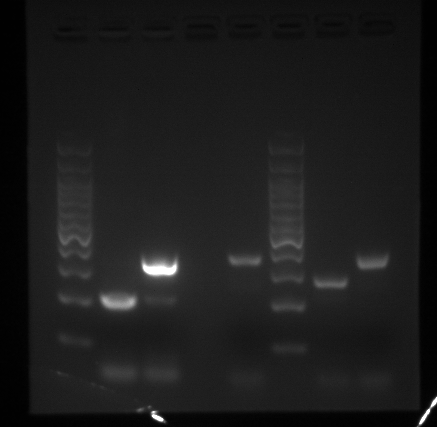

Supplement: Figure 1—figure supplement 2—source data 1. [file elife-94754-fig1-figsupp2-data1.zip › Figure 1—figure supplement 2—source data 1/panel E/E.tif]

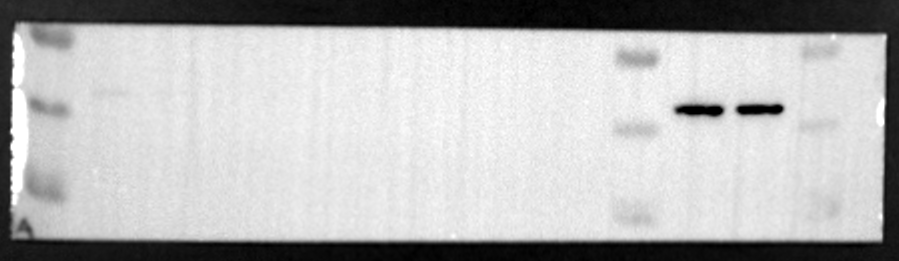

Supplement: Figure 1—figure supplement 2—source data 1. [file elife-94754-fig1-figsupp2-data1.zip › Figure 1—figure supplement 2—source data 1/panel F/beta-actin.tif]

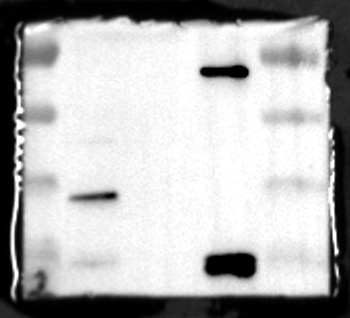

Supplement: Figure 1—figure supplement 2—source data 1. [file elife-94754-fig1-figsupp2-data1.zip › Figure 1—figure supplement 2—source data 1/panel F/Xap5.tif]

Figure 1—figure supplement 2

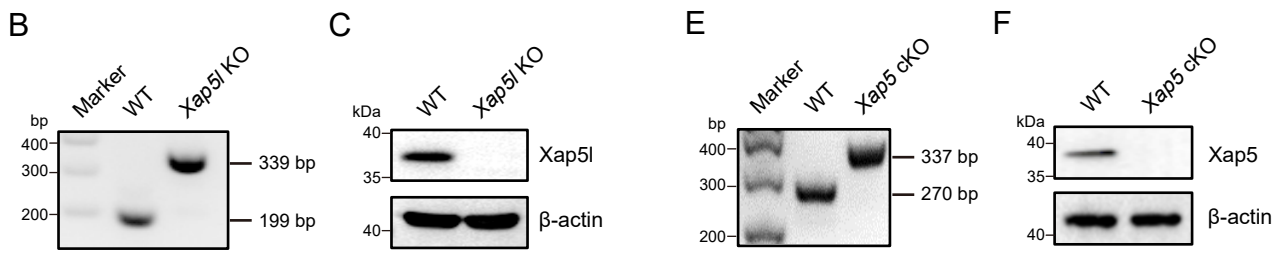

Raw blots:

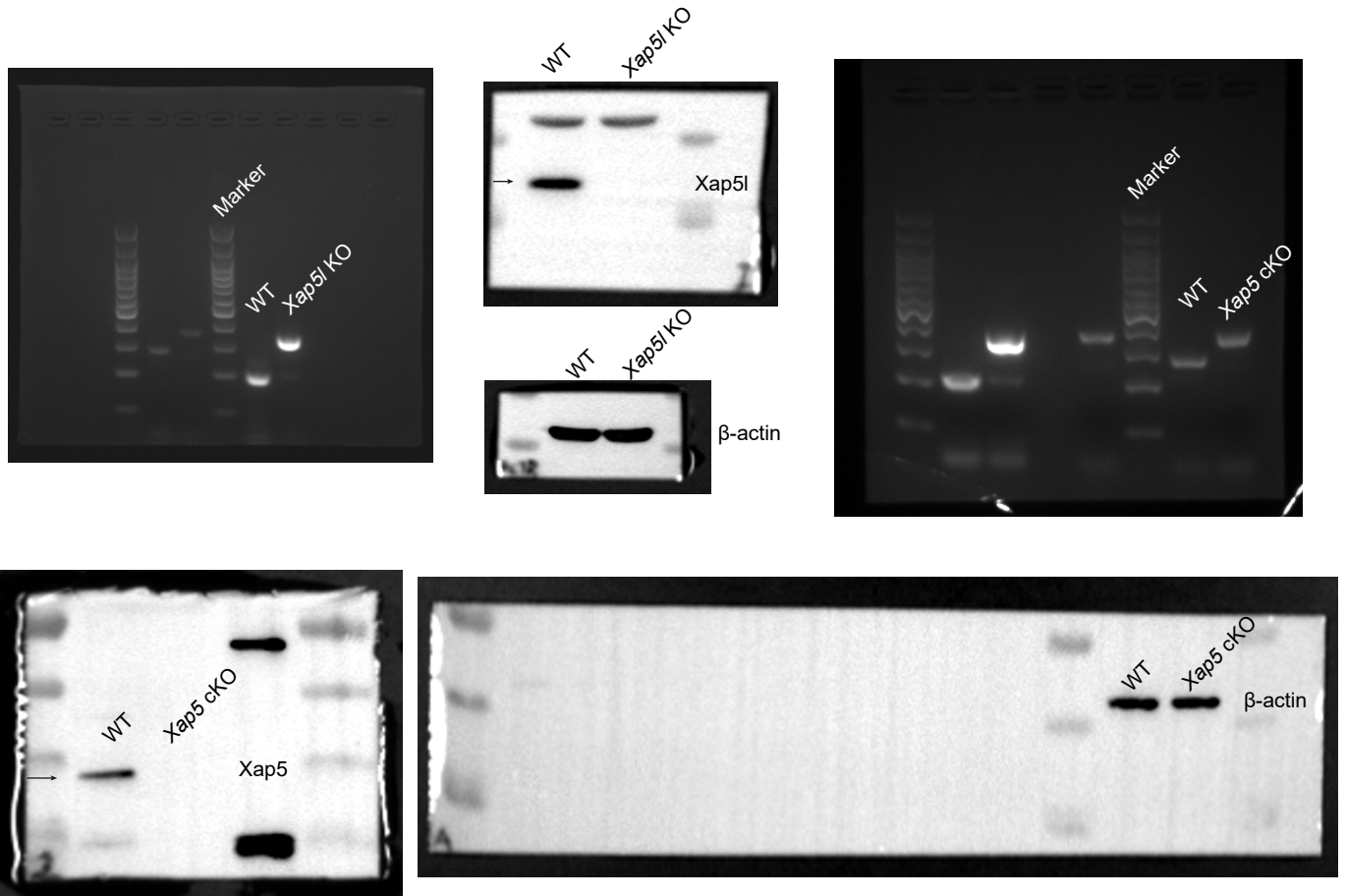

Supplement: Figure 1—figure supplement 2—source data 2. [file elife-94754-fig1-figsupp2-data2.zip › Figure 1—figure supplement 2—source data 2/Figure 1—figure supplement 1—source data 2.pdf]

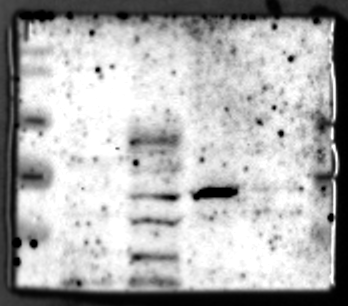

Supplement: Figure 4—source data 1. [file elife-94754-fig4-data1.zip › Figure 4—source data 1/panel C/Foxj1.tif]

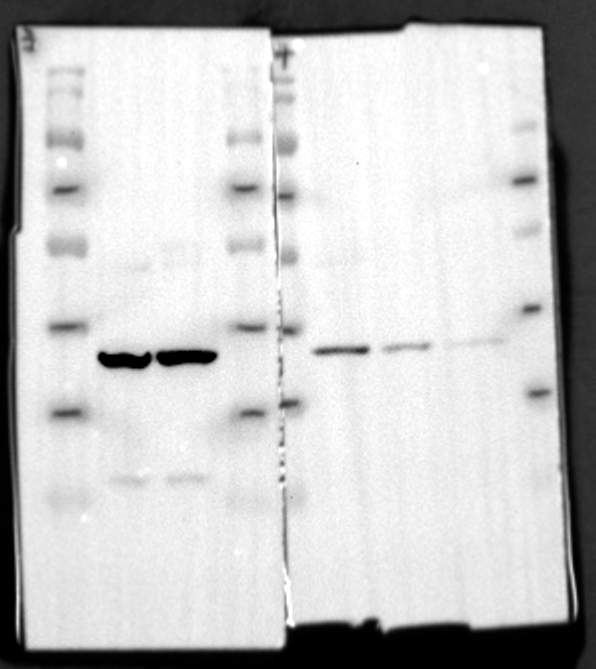

Supplement: Figure 4—source data 1. [file elife-94754-fig4-data1.zip › Figure 4—source data 1/panel C/Gapdh@Foxj1.tif]

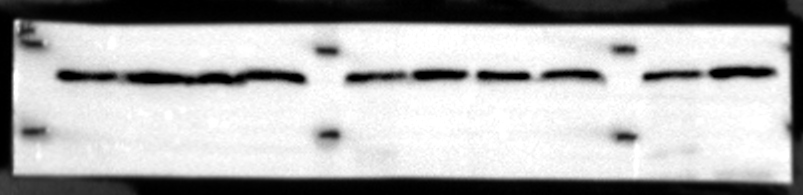

Supplement: Figure 4—source data 1. [file elife-94754-fig4-data1.zip › Figure 4—source data 1/panel C/Gapdh@Rfx2.tif]

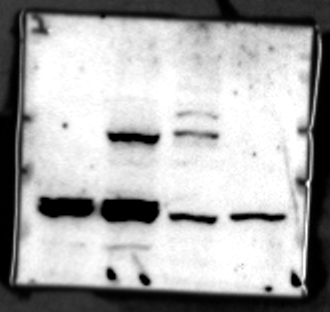

Supplement: Figure 4—source data 1. [file elife-94754-fig4-data1.zip › Figure 4—source data 1/panel C/Rfx2.tif]

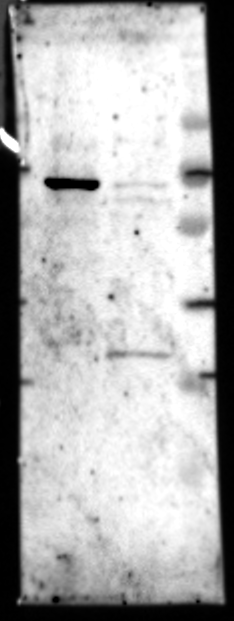

Supplement: Figure 4—source data 1. [file elife-94754-fig4-data1.zip › Figure 4—source data 1/panel F/Foxj1.tif]

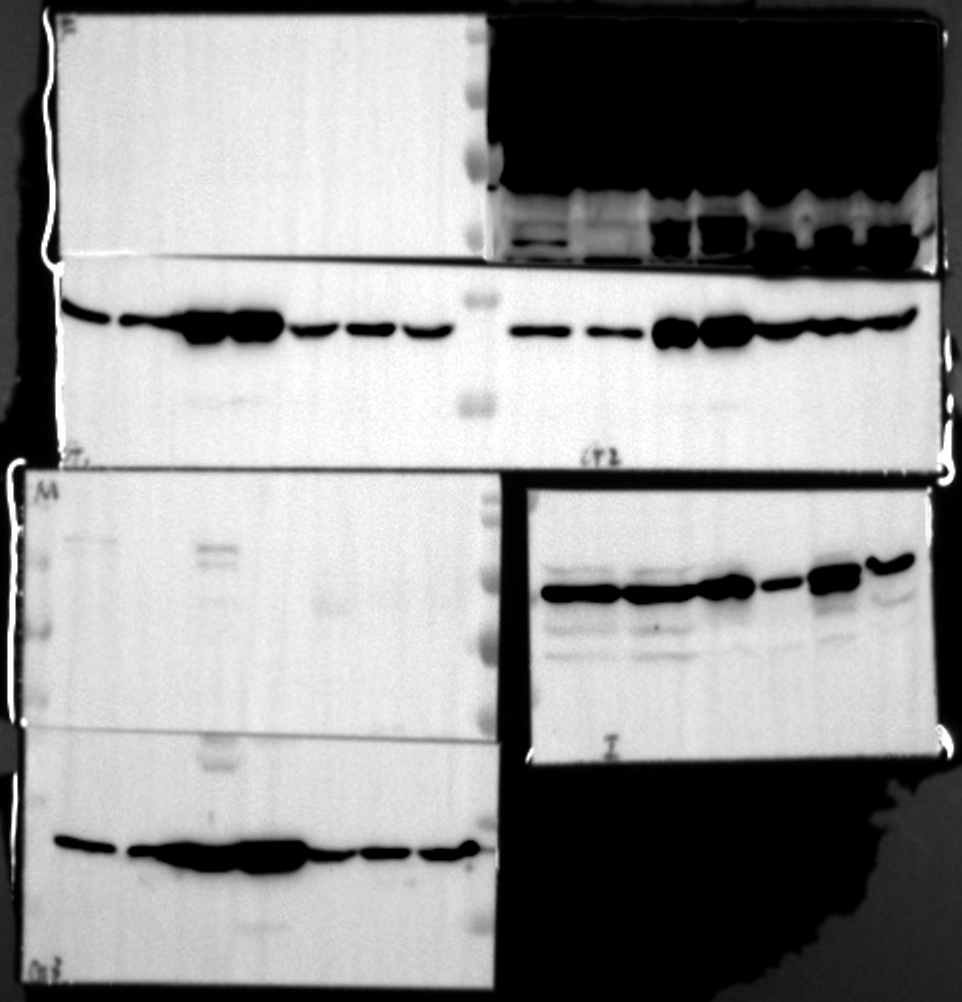

Supplement: Figure 4—source data 1. [file elife-94754-fig4-data1.zip › Figure 4—source data 1/panel F/Gapdh@Foxj1.tif]

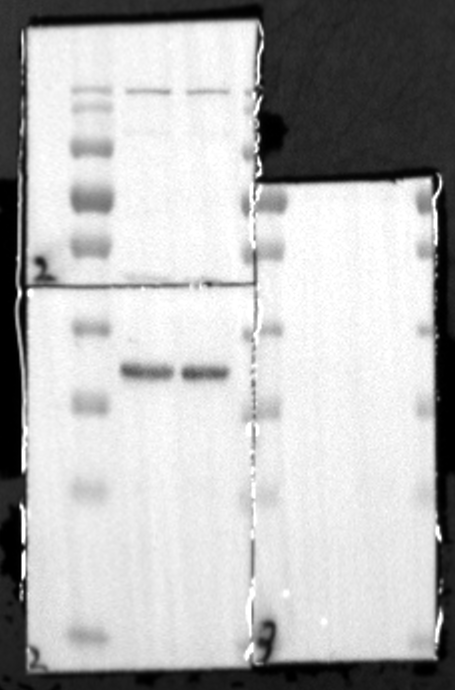

Supplement: Figure 4—source data 1. [file elife-94754-fig4-data1.zip › Figure 4—source data 1/panel F/Gapdh@Rfx2.tif]

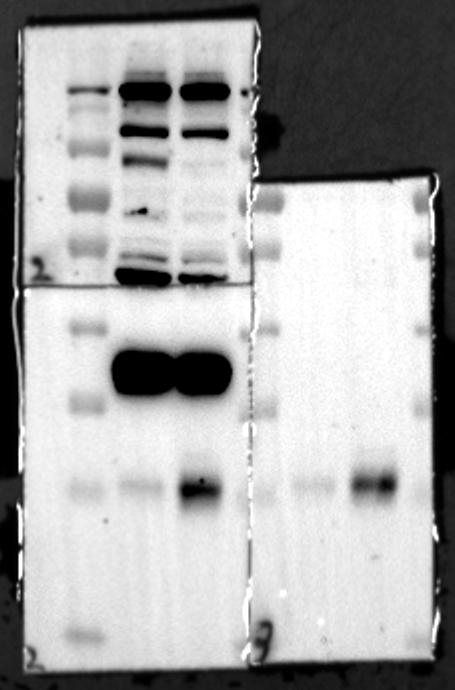

Supplement: Figure 4—source data 1. [file elife-94754-fig4-data1.zip › Figure 4—source data 1/panel F/Rfx2.tif]

Figure 4

C

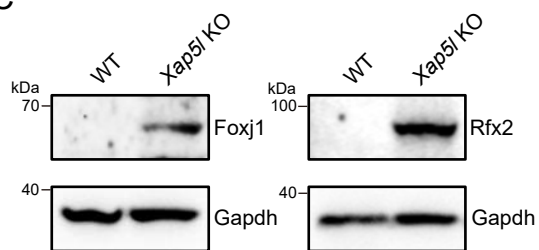

Raw blots:

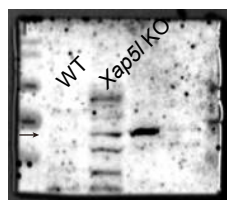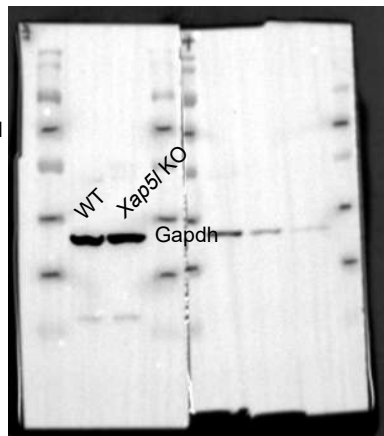

F

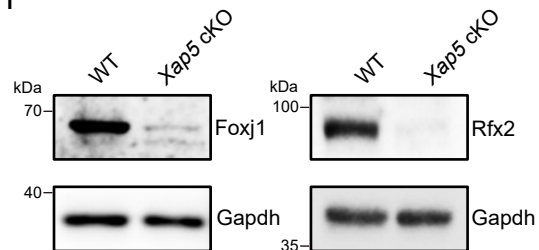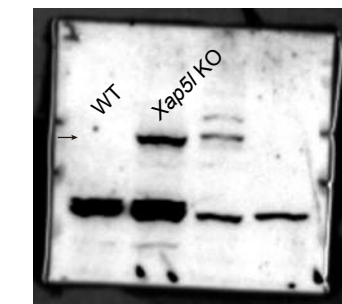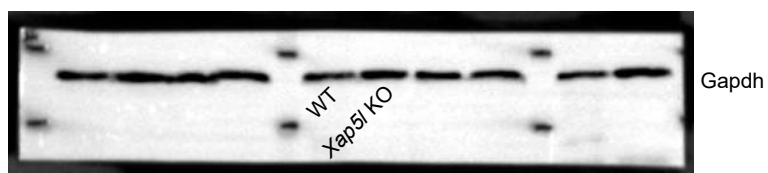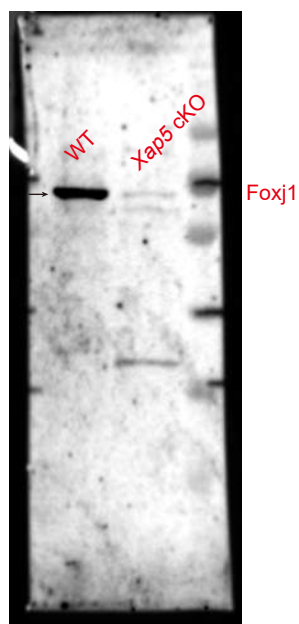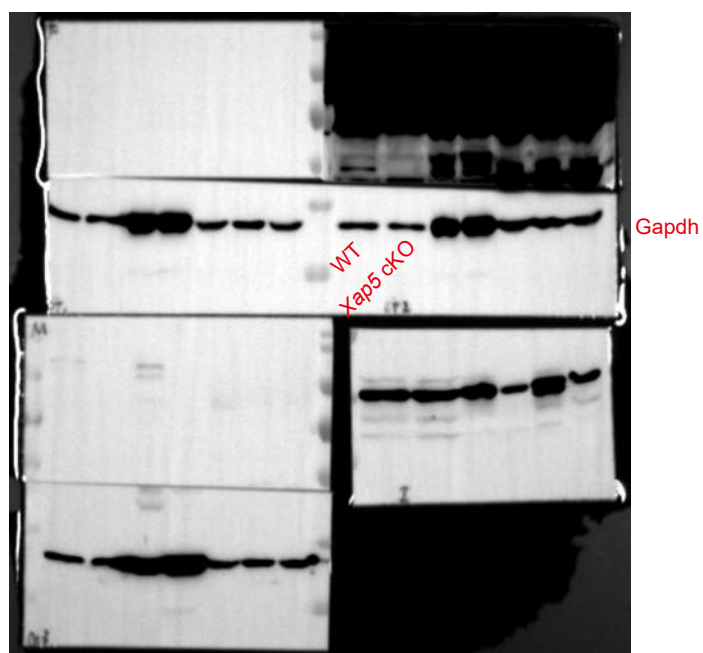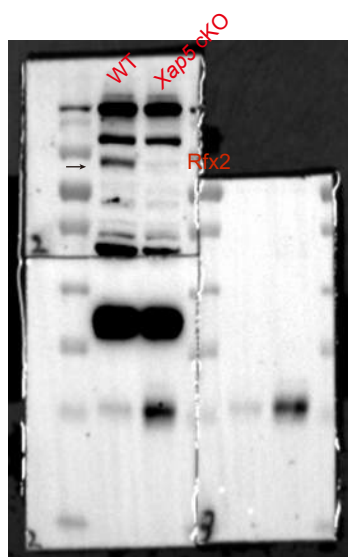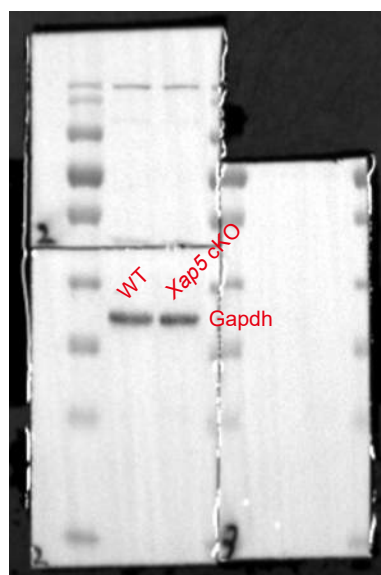

Supplement: Figure 4—source data 2. [file elife-94754-fig4-data2.zip › Figure 4—source data 2/Figure 4—source data 2.pdf]

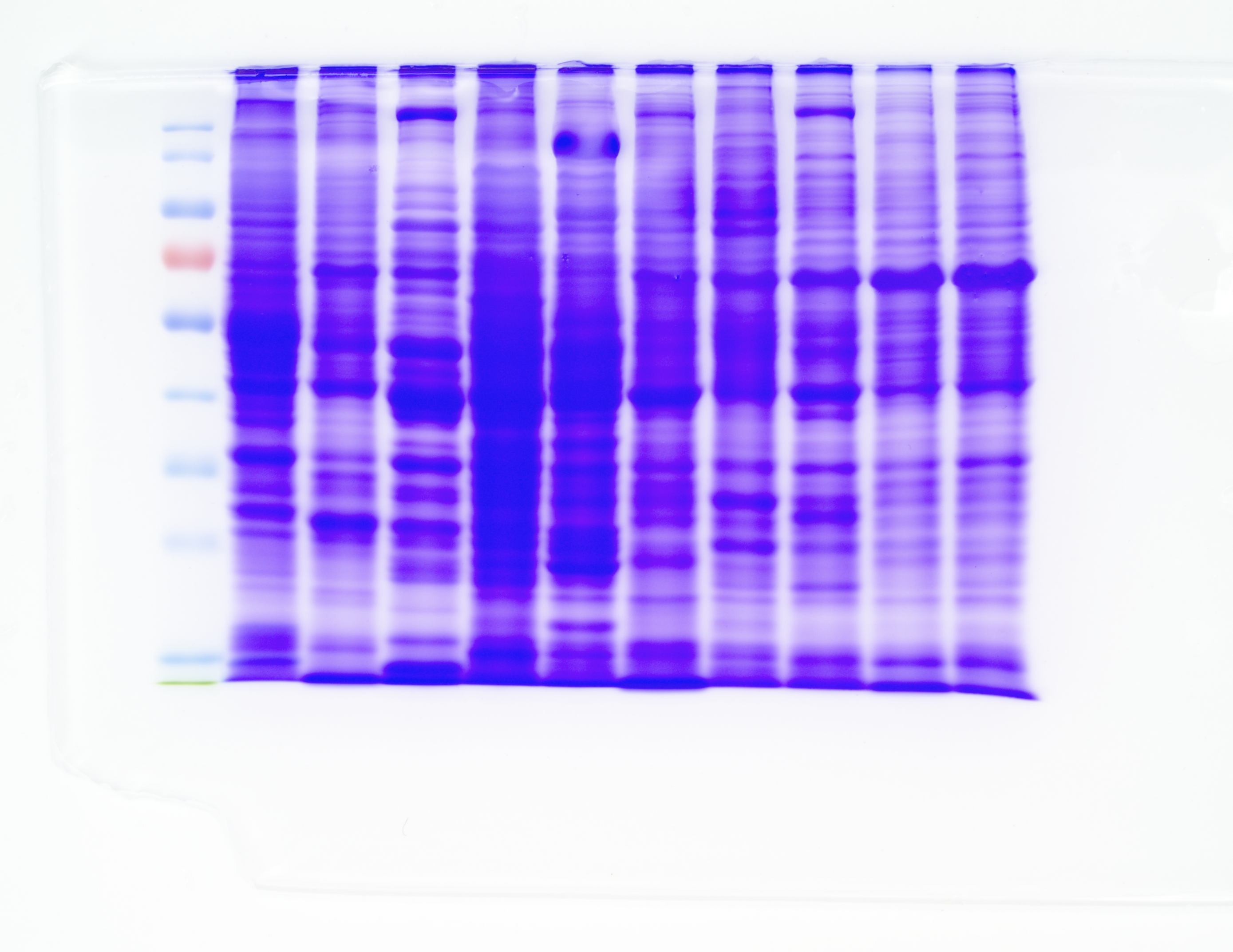

Supplement: Figure 4—figure supplement 2—source data 1. [file elife-94754-fig4-figsupp2-data1.zip › Figure 4—figure supplement 2—source data 1/CBB staining.tif]

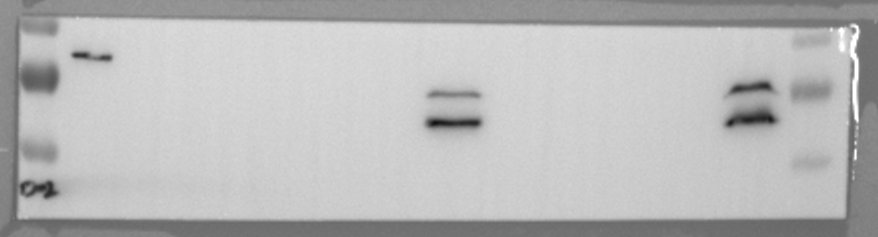

Supplement: Figure 4—figure supplement 2—source data 1. [file elife-94754-fig4-figsupp2-data1.zip › Figure 4—figure supplement 2—source data 1/Tulp2.tif]

Figure 4—figure supplement 2A

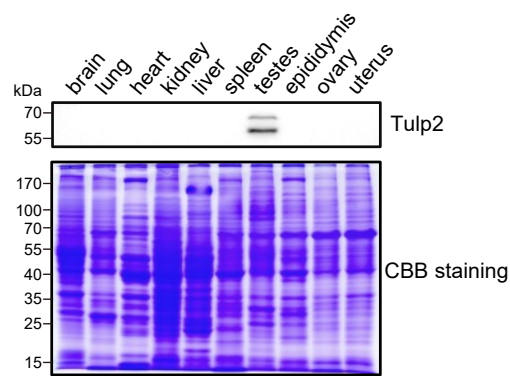

Raw blots:

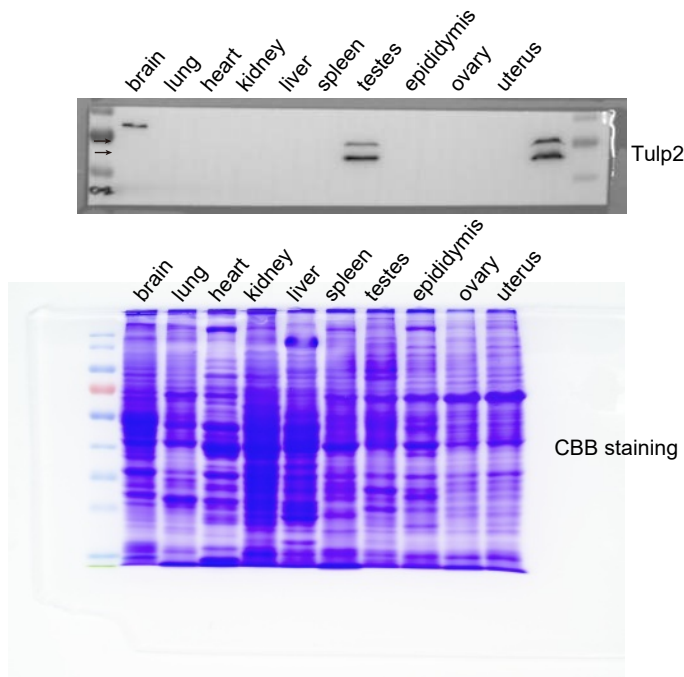

Supplement: Figure 4—figure supplement 2—source data 2. [file elife-94754-fig4-figsupp2-data2.zip › Figure 4—figure supplement 2—source data 2/Figure 4—figure supplement 2—source data 2.pdf]
